# Neuroinflammation: Microglia and T Cells Get Ready to Tango

**DOI:** 10.3389/fimmu.2017.01905

**Published:** 2018-01-25

**Authors:** Sjoerd T. T. Schetters, Diego Gomez-Nicola, Juan J. Garcia-Vallejo, Yvette Van Kooyk

**Affiliations:** ^1^Department of Molecular Cell Biology and Immunology, VU University Medical Center, Amsterdam, Netherlands; ^2^Centre for Biological Sciences, University of Southampton, Southampton, United Kingdom

**Keywords:** T cells, microglia, tolerance, central nervous system, antigen presentation, inflammation

## Abstract

In recent years, many paradigms concerning central nervous system (CNS) immunology have been challenged and shifted, including the discovery of CNS-draining lymphatic vessels, the origin and functional diversity of microglia, the impact of T cells on CNS immunological homeostasis and the role of neuroinflammation in neurodegenerative diseases. In parallel, antigen presentation outside the CNS has revealed the vital role of antigen-presenting cells in maintaining tolerance toward self-proteins, thwarting auto-immunity. Here, we review recent findings that unite these shifted paradigms of microglial functioning, antigen presentation, and CNS-directed T cell activation, focusing on common neurodegenerative diseases. It provides an important update on CNS adaptive immunity, novel targets, and a concept of the microglia T-cell equilibrium.

## Highlights

- Microglia represent the main antigen-presenting cell (APC) inside the brain parenchyma during neurodegeneration.
- MHCII^+^ microglia and CD4^+^ T cells accumulate during chronic neurodegeneration and reciprocally shape pathology.
- Brain tissue and microglia transcriptomics show upregulation of genes, involved in APC–T cell interactions, that is shared across neurodegenerative diseases.
- Current preclinical treatment strategies affect the equilibrium between microglia and T cells.

## Introduction

In the central nervous system (CNS), innate immune surveillance is mainly coordinated by the brain’s resident myeloid cell, the microglia. Microglia are derived from erythro-myeloid progenitors residing in the yolk-sac before embryonic day 8 and are not replenished by blood-derived monocytes under physiological conditions (1, 2). Instead, they are able to continuously proliferate to maintain a stable population throughout life (3). Microglia react to counteract any disturbances in immunological homeostasis to protect neurons with a limited capacity to regenerate. The activation and proliferation of microglia is highly increased during neurodegeneration, including Alzheimer’s disease (AD), Parkinson’s disease (PD), amyotrophic lateral sclerosis (ALS), multiple sclerosis (MS), and prion-induced neurodegeneration (4). Commonalities in the neuro-inflammatory response in these diseases involve modest T cell infiltration, microglial proliferation and activation, accumulation of misfolded self-antigens and progressive neuronal dysfunction, and death. A crucial effector element of the adaptive immune system, the T cell, has been known for its destructive role in MS. Recently, however, CNS-infiltrating T cells have been reported to function in limiting neuronal damage caused by infection, mechanical injury, or neurodegenerative disease (5). What regulates this balance between neuroprotection and neurodestruction is incompletely understood. The initial activation of a CNS-reactive T cell responses from naïve T cells is generated outside the brain (6), and microglia are not considered as dendritic cells (DCs) by current definitions (i.e., uptake and processing of antigen and the subsequent migration to draining lymph nodes for antigen presentation to naïve T cells; see Box 1). However, during neurodegenerative disease, the microglial population is the main major histocompatibility complex (MHC) class II-expressing antigen presenting cell (APC) in the brain parenchyma, where neuronal damage can be found. Interestingly, even though the accumulation of MHCII^+^ microglia and reactive CD4^+^ T cells is common during neurodegeneration, the interaction of these two cell types at places of neurodegeneration remains undefined. Here, we review recent studies that provide evidence for involvement of both T cell and microglia as a response to neurodegenerative disease. We delineate the concept of MHC class II-mediated antigen presentation during chronic neurodegeneration, with a focus on MHCII^+^ microglia and the reactivation of CNS-specific T cells in the brain parenchyma. Next, we explore the hypothesis of microglia–T cell interactions and consider their phenotype as a result of reciprocal signaling. Finally, we propose a model of the hypothesized microglia T-cell equilibrium.

Box 1. Initiation of adaptive immunity and homeostasis toward the central nervous system (CNS). While the CNS was once thought to be an immune privileged site, a defined lymphatic and glympathic system is able to drain CNS-derived antigens and mount CNS-directed adaptive immune responses (6, 7). As in other peripheral organs, acquired tolerance to self-antigens is paramount in preventing auto-immunity. The initiation of T cell-mediated adaptive immune responses against CNS-derived antigens relies mostly on antigen drainage to DCs in adjoining structures like the choroid plexus, the leptomeningeal spaces, and the deep cervical lymph nodes (6). It is also through these barriers that CNS immune surveillance and T cell infiltration occurs (5, 7). Encephalitogenic T cells can be primed to enter the CNS parenchyma by leptomeningeal phagocytes, lung-resident DCs, and choroid plexus APCs (6). These cells are derived from monocytic precursors and actively present CNS-derived self-antigens to CD4^+^ T cells in homeostatic conditions (8). As a result, T cells are able to infiltrate the CNS and affect immunological equilibrium in both a pathogenic and supportive manner (5). In the presence of immunological disturbance in the CNS, T cells can be neuro-protective on the short term but can become pathogenic when inflammation is not resolved. For example, it has been shown that an acute inflammatory response after neuronal damage elicits a protective T cell response. Neuroprotection relied on CNS antigen-specific CD4^+^ T cells generated in the draining lymph nodes (9, 10). In turn, this response is subsequently balanced by regulatory T cells to prevent auto-immunity (10). Interestingly, regulatory T cells pre-exposed to microglia were able to reduce neuronal death in organotypic hippocampal slices (11). A more recent study has shown a direct remyelinating effect of regulatory T cells after neuronal damage by promoting oligodendrocyte progenitor cell differentiation and myelination (12). In contrast, regulatory T cell can also exacerbate neuronal loss by affecting CNS-resident macrophage/microglia phenotype in an antigen-independent manner (13). As such, phenotyping the CD4^+^ T cells in the brain may be insufficient to explain disease outcome and indirect effects of T cells during pathology through accessory cells should be taken into account. Thus, while a well-controlled CD4^+^ T cell response can promote neuronal survival and recovery, sustained T cell activation or hyper activation can become pathogenic and balancing the response through induction of regulatory T cells seems to prevent or even repair CNS damage.

## Chronic Neurodegeneration Involves the Infiltration of T Cells

Pathogenic T cells are well described in CNS autoimmunity like MS and experimental autoimmune encephalomyelitis (EAE), the mouse model of inflammatory demyelination. While the causes for MS remain to be identified, effector T cells specific for myelin constituents induce inflammation upon recognition of the myelin sheaths, leading to neuronal dysfunction and death. In EAE, the pathology depends mostly on CD4^+^ T cells and their phenotype is mostly Th1 and Th17, reflecting the pro-inflammatory pathology of MS. At the same time, regulatory T cells seem to be defective and incapable of suppressing effector T cells in EAE (14). Boosting their suppressive functionality has therefore been proposed as a therapeutic strategy in MS (15). While MS and EAE are considered to be CNS antigen-specific autoimmune disorders with intra-parenchymal antigen presentation, the antigens that are presented at sites of T cell infiltration remain unknown. Regardless, it is clear that T cell activation in the case of MS and EAE represents a highly pathogenic form of CNS autoimmunity. Less obvious is the role of T cells in chronic neurodegenerative diseases, like PD, AD, ALS, and prion-induced neurodegeneration, mainly because overt T cell activation and clonal expansion is limited and never reaches levels seen in EAE and MS. Nonetheless, evidence is steadily mounting that T cells play a common role in shaping neurodegenerative diseases.

Increased T cell infiltration has been found in postmortem brain tissue of PD patients (16). Importantly, a recent study has shown that CD4^+^ T cells from PD patients specifically react to antigenic MHC class II epitopes derived from α-synuclein (17). Several studies have therefore tried to investigate which type of T cell is involved, which function it performs and how it affects pathogenesis. Mouse models for PD can be either toxin-induced (i.e., MPTP neurotoxicity) or through viral overexpression of α-synuclein. The MPTP toxin kills dopaminergic neurons and destroys the dopaminergic nigrostriatal pathway, mimicking PD pathology. MPTP-induced neurotoxic mice lacking T cells, especially CD4^+^ T cells, showed reduced dopaminergic cell death and microglial activation (16). Alternatively, viral overexpression of full length α-synuclein in the mouse brain leads to progressive neurodegeneration of dopaminergic neurons. In this model of PD, MHCII is highly upregulated on microglia and the genetic loss of MHCII (and CD4^+^ T cells) reduced α-synuclein induced microglial activation and the degeneration of dopaminergic neurons (18). Therapeutically, boosting the function or quantity of regulatory T cells reduced pathology, in part by reducing microglial activation (19). These data suggest that CD4^+^ T cells affect PD disease pathology, potentially through MHC class II-expressing parenchymal microglia.

Of all forms of dementia, AD is the most prevalent form, characterized by accumulation of the self-antigen amyloid β, widespread microglial activation, and neuronal loss. Increased T cell infiltration has also been shown in brains of AD patients (20) and T cells in the afflicted hippocampal region are located in close proximity to microglia (21). Peripheral T cells from human patients demonstrated increased reactivity to amyloid β (22). In contrast, the phenotype of peripheral T cells in transgenic AD mice has been reported to be regulatory or suppressive (23) and hypo-responsiveness of CD4^+^ T cells from AD transgenic mice after re-stimulation suggests T cell tolerance to the antigen (24). These data demonstrate that discrepancies exist between adaptive immune regulation in human AD patients and mouse models of the disease. An intriguing recent set of publications demonstrated the involvement of regulatory T cells in dampening the neuro-protective “auto-immune” T cell response in AD transgenic mice and that blocking this response attenuates AD-like pathology (23, 25). Of note, these studies are limited by the focus of the peripheral responses, and it remains to be investigated whether peripheral and CNS-resident T cells are functionally different. Nonetheless, these data suggest the involvement of early pathogenic tolerance in AD transgenic mice; a notion that is paralleled by suppressive microglial phenotypes (discussed below).

In the blood and spinal cord of patients with ALS, CD4^+^ T cells are increased (26) with a predominantly pro-inflammatory Th1/Th17 phenotype (27). Regulatory T cells from blood of ALS patients demonstrated a significant decrease in the ability to suppress proliferation in effector T cells and the extent of loss in suppression was correlated with disease progression (28). The chronic degeneration of motor neurons and muscle weakness seen in ALS patients can be modeled in transgenic mice harboring the human superoxide dismutase 1 (SOD1) mutation (G93A). CD4-knockout SOD1 transgenic mice showed increased disease progression, suggesting a protective role for CD4^+^ T cells on pathology (29). Interestingly, infusion of both regulatory- and effector T cells delayed loss of motor function and extended survival. In this study, Tregs delayed the onset of pathological symptoms, while effector T cells increased the latency from initiation to terminal phases (30). Similarly, regulatory T cells may dominate during the slow progressing phase, while loss of suppression and pro-inflammatory T cells dictates the rapidly progressing phase (31). Hence, a time-dependent disequilibrium of the adaptive immune response may occur during ALS, a concept that seems to be paralleled by infiltration of monocytes in the peripheral nervous system that precedes CNS pathology in this model (32).

Lastly, Creutzfeldt–Jakob (Prion) disease is a transmissible infectious CNS disease that is characterized by progressive neuronal loss, aggregation of a misfolded self-protein (PrP^sc^), microglial activation, and T cell infiltration (33, 34). Because of the low incidence of Creutzfeldt–Jakob disease, few studies investigate T cell functioning in patients and instead rely on murine models of scrapie. In mice, fragments of the prion protein can be presented in murine MHC class II and activate CD4^+^ T cells (35). Interestingly, prion-specific T cells invade the prion-infected mouse brain but are dysfunctional, showing lack of lytic function or secretion of pro-inflammatory cytokines like IFNγ and TNFα (34). Hence, while prion-specific T cells may be generated and infiltrate the brain, they do not produce pro-inflammatory signals and can instead be considered inactivated at the site of cellular damage.

It is clear that T cell numbers increase in multiple murine models of chronic neurodegeneration and their phenotype changes during disease progression and specific pathological phases. However, in human forms of these diseases, the balance of tolerance and inflammation is still incompletely understood. In this regard, stimulating regulatory function of infiltrating CD4^+^ T cells may act therapeutically in MS, PD and to a certain degree in ALS, while in AD and prion disease CD4^+^ T cells already seem regulatory and may benefit from a more pro-inflammatory T cell phenotype. This indicates that T cell infiltration and dysfunction is a common denominator in models of chronic neurodegeneration, but how are effector T cell responses against CNS-antigens coordinated?

## MHC Class II is Linked to the Inflammatory Response in Neurodegenerative Diseases

Effector T cells are coordinated by APCs both at the initiation phase in secondary lymphoid organs and at the effector phase at the site of inflammation. MHC class I (MHCI) and MHC class II (MHCII) enable antigen presentation to CD8^+^ T cells and CD4^+^ T cells, respectively. MHCI is expressed by all nucleated cells, while MHCII complexes are only expressed by APCs, such as DCs, B cells, and microglia. While expression of MHCII is low in homeostatic conditions in the brain, it can be rapidly upregulated on microglia and is often used as a marker of their activation (36). Since empty MHCII complexes are quickly degraded before they reach the plasma membrane, the widespread membrane expression of MHCII on microglia is evidence for its potential to present antigen. Still, there have been no reports on microglia migrating outside the brain to draining lymph nodes for the activation of naïve T cells. Instead, monocyte-derived DCs located at neighboring sites around the brain like the deep cervical lymph nodes capture draining antigens for T cell responses directed against CNS-borne antigen (see Box 1). It is therefore more likely microglia present antigen on MHCII for reactivation and modulation of incoming effector CD4^+^ T cells.

Major histocompatibility complex class II (encoded in humans by the HLA-DR genes) has been linked to many neurodegenerative diseases. An extensive meta-analysis of genome-wide association studies (GWAS) in individuals of European ancestry revealed HLA-DRB5 and HLA-DRB1 as susceptibility loci for late-onset AD (37). Similarly, GWAS studies show association of the HLA-DR genetic region with PD (38) and MS (39). In AD patients, MHC II is upregulated and expressed by microglia (40) and is conversely correlated with cognitive ability (41). AD post-mortem brain tissue transcriptomics revealed significant upregulation of MHCII antigen processing and presentation machinery in the later stages of AD pathology (42). In afflicted brain areas of PD and MS patients, MHCII is highly expressed on microglia (20). Recently, a comparative transcriptomics study of brain tissue of AD, ALS, PD, and MS patients showed shared upregulation of genes involved in antigen presentation, including HLA-DR, compared to age-matched controls (43). These data suggest that (1) HLA-DR genetic predisposition for certain neurodegenerative diseases implicates antigen-specificity in the context of MHCII as a driver of disease and (2) MHC II expression by microglia is a shared feature of the neuroinflammatory response in common neurodegenerative diseases. When effector CD4^+^ T cells exit the secondary lymphoid organs, it is assumed they do not exert their function randomly in the circulation. Instead, a “second touch” may be provided when they recognize their cognate antigen in association with MHCII at the target tissue (44). Whether the antigen presented by MHCII^+^ microglia is immunogenic for reactive CD4^+^ T cells during neurodegeneration remains unknown.

## Microglia Share Machinery Needed for Antigen Recognition, Uptake, and Presentation with Peripheral APCs

Microglia are seldom dormant and constantly sample the environment, including neurons, astrocytes, and other cell types. As the brain-resident macrophage, microglia are equipped with the tools to properly recognize and react to changes in the environment. Apart from toll-like receptors (TLRs), which have been expertly reviewed elsewhere (45), microglia express many pattern recognition receptors (PRRs) that bind and internalize foreign or misfolded proteins. Often, these molecules are upregulated in the presence of neuronal damage or inflammation (4). For example, triggering receptor expressed on myeloid cells-2 (TREM2) on microglia binds and takes up anionic lipids, a lipid form that associates with the toxic fibrillar amyloid-β, and APOE in AD patients (46, 47). TREM2 also binds phosphotidylserine, a phospholipid that is shuttled to outside of the plasma membrane in cells undergoing apoptosis, including degenerating neurons (46). In a recent elegant study, Krasemann and colleagues have demonstrated a Trem2-Apoe pathway that instructs a disease-associated microglial phenotype after phagocytosis of apoptotic neurons (48). TREM2 also provides an immune suppressive signal upon binding *via* the intracellular DAP12 in microglia, which in DCs results in reduced capacity to activate T cells (49). Since neuronal damage like demyelination may result in unrepairable loss of function, microglia are quick to react to changes in neurons. Glycosylation plays a role in the protection of neurons through sialic acids, the capping monosaccharides on glycans. The interaction of neuronal sialic acids with CD33/Siglec-3 on microglia releases an inhibitory signal, which has been implicated in AD (50). However, when sialic acids on neurons are lost, neurites can be “tagged” by C1q and taken up by microglial complement receptor 3 (CR3), another PRR that signals through DAP12. CR3 additionally binds iC3b which covalently binds weak synapses and debris as a signal for elimination and phagocytosis (51). In DCs, CR3 activation leads to reduced capacity to stimulate antigen-specific T cells (52). Even more, intraocular tolerance induction depends on IL10 and TGFβ production after ligation of iC3b to CR3 on APCs (53). The DAP12 signaling molecule relays signals from several microglial receptors and has been implicated as a key causal regulator of late-onset AD (54). Another mechanism that involves microglial sampling of neurons is the glycosylation of myelin. Normally, fucosylated myelin is recognized by microglial DC-SIGN, internalized and presented to T cells, reducing T cell activation (55). In MS, fucose is removed from the myelin and escapes DC-SIGN-mediated suppression leading to increased T cell proliferation and differentiation toward the pathogenic Th17 T cell phenotype (55). Furthermore, microglial activation is additionally suppressed by neurons through CD200R and CX3CR1, since loss of this interaction results in aberrantly activated microglia (4).

**Figure 1:**
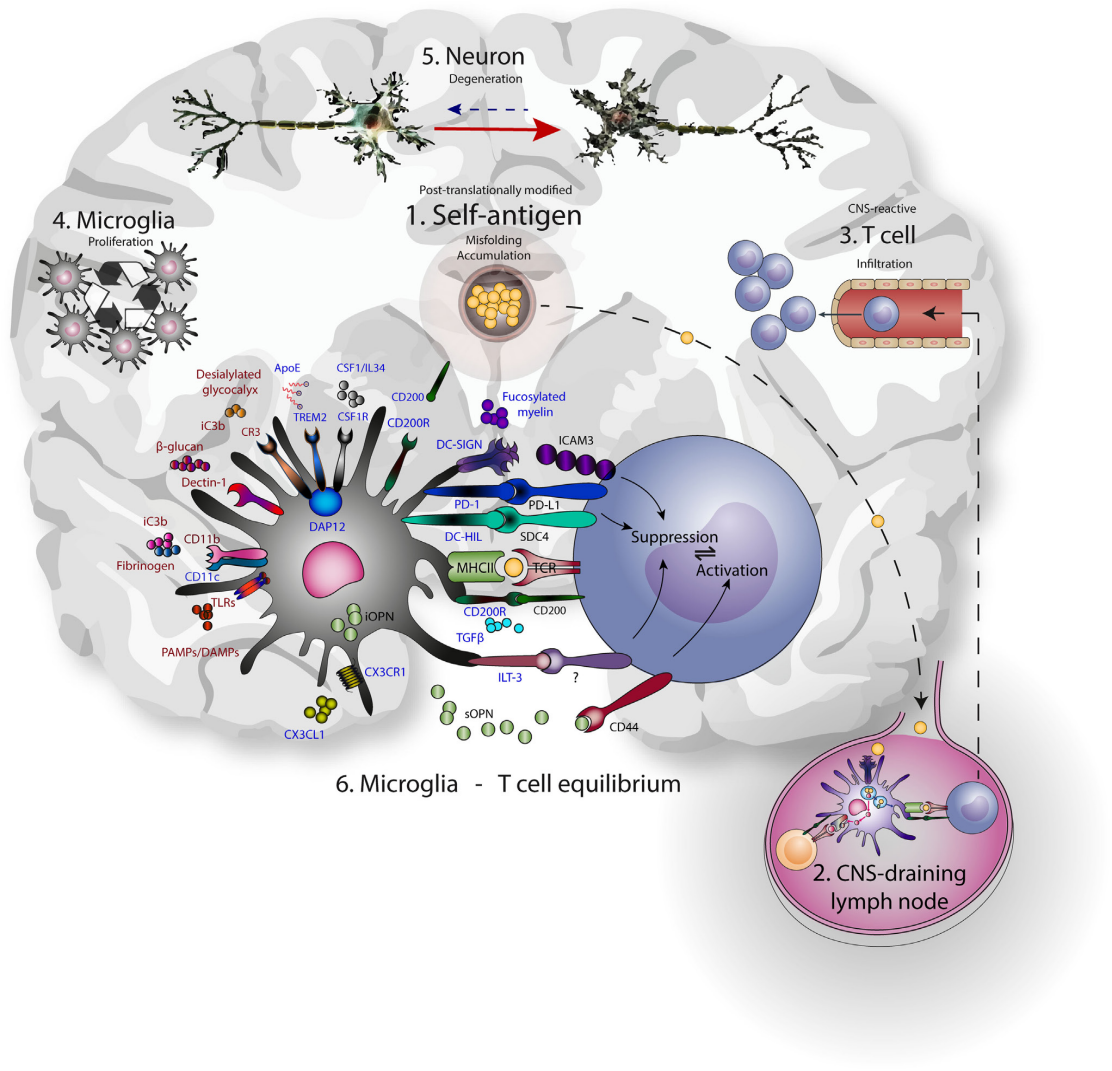
Microglia-T cell equilibrium; activated microglia and infiltrating T cells interact during chronic neurodegeneration and shape central nervous system (CNS) immunology and neuropathology. Commonalities of neurodegenerative disease include accumulation of misfolded self-antigen, T cell infiltration, microglial proliferation and activation, and progressive neuronal dysfunction and death. (1) Self-antigens like amyloid β and α-synuclein are accumulating and often aberrantly post-translationally modified. (2) These antigens can drain to lymph nodes outside the CNS and be presented to T cells by resident antigen-presenting cells. (3) CNS-antigen-specific T cells enter the brain through the vasculature and encounter microglia in the brain paranchyma. (4) Microglia express many molecules that recognize and bind neuronal aberrancies (5) like desialylated glycocalyx or complement deposition on neurites, as well as aberrant self-antigens like aggregated amyloid β. At the same time, these receptors induce proliferation and signaling. Inhibitory receptors (represented in blue) are mainly implicated in reducing inflammation, while the activating receptors (represented in red) mainly induce inflammation. As a result, cellular debris and antigen is taken up, processed, and presented on MHC class II, enabling the interaction with infiltrating antigen-specific T cells. In turn, contact-dependent interactions and soluble factors may affect the phenotype of incoming T cells. (6) The microglia-T cell equilibrium ensures limitation of protective adaptive immunity after neuronal damage and prevents auto-immunity toward CNS-derived antigens.

Hence, microglia use PRRs to sample and control the environment, including neurons, and act accordingly to prevent excessive damage. These same PRRs are often used by peripheral DCs and macrophages for the uptake and presentation of antigen to T cells. Additionally, CR3, TREM2, CD200R, CX3CR1, and DC-SIGN suppress microglia and APC-mediated T cell activation, with a central role assigned to DAP12. The expression of this multitude of molecules linked to antigen presentation on microglia reveals a delicate interplay of neuronal, microglial, and T cell interactions aimed at reducing excessive neuronal damage (Figure 1).

## Microglia in Neurodegenerative Diseases and Primary Brain Tumors Highly Express Genes Involved in T Cell Modulation

Traditionally, the analysis of microglial phenotypes was based on M1 and M2 (pro- and anti-inflammatory, respectively) markers, biasing and hampering research progress (56). A closer look at transcriptomes from brain tissue and isolated microglia from neurodegenerative diseases identifies a set of genes highly upregulated in microglia that is shared between the diseases (see Table 1). These genes are among the highest upregulated genes in these models and encode proteins that are known to be highly expressed by DCs in the peripheral immune system, affecting T cell functioning. Surprisingly, their role in the CNS has been ill defined, especially considering their widespread overexpression in murine models of neurodegeneration.

**Table 1:** Shared upregulated genes in murine and human tissue/microglia across central nervous system afflictions.

	Murine models of chronic neurodegeneration							Human AD	Murine glioblastoma	Human glioblastoma
		
	Total cortex			Acutely isolated microglia				Cortex	Acutely isolated microglia	Tumor tissue
			
	ME7 Prion	APP/PS1 AD	5xFamilial AD	APP/PS1 AD end-stage	APP/PS1 AD CD11c^+^	CVN AD CD11c^+^	SOD1 g93a ALS Spinal cord	GL261 glioblastoma		
CD11c/*Itgax*	+++	++	++	+	+	+++	++	ND	+	+
Dectin-1/*Clec7a*	+++	++	++	+	+		++	+	+	+
DC-HIL/*Gpnmb*	++	++	+	+++	++	+++	+++	+	++	+
PD-1/*Pdcd1*	++	++	+	+	+	+++	+	ND		+
OPN/*Spp1*	++	+		+	++	+++	+++	+	++	+
ILT3/*Lilrb4*	++	+	+	+	+	+++	++	+	+	+

*A comparison of transcriptomic studies reveals shared upregulated genes in microglia across multiple neurodegenerative diseases. ME7 Prion (57) (compared to normal brain homogenate), APPswePS1dE9 (APP/PS1) (58) (compared to age-matched control), 5XFAD (59) (compared to age-matched control), APP/PS1 end-stage microglia (60) (compared to age-matched WT controls), APP/PS1 CD11c^+^ microglia (61) (compared to CD11c^−^ microglia), CVN AD CD11c^+^ microglia (62) (compared to CVN AD CD11c^−^ microglia), SOD1 g93a ALS spinal cord microglia (63) (compared to age-matched WT controls), Human AD (64) (compared to age-matched non-demented controls), GL261 glioblastoma microglia (65) (compared to age-matched WT microglia), Human glioblastoma tissue (TCGA database; compared to non-diseased tissue); ND, not determined; Blank, unknown. += > 2-fold, ++ = > 10-fold, +++ = > 50-fold*.

Dendritic cell-HIL (encoded by *Gpnmb*), ILT3 (encoded by *Lilrb4*), and PD-1 (encoded by *Pdcd1*) are mainly described as immunosuppressive modulators of adaptive immunity. For example, DC-HIL inhibits T cell receptor (TCR)-dependent proliferation of T cells and blocks reactivation of T cells previously activated by APCs (66). Among APCs, DC-HIL expression is the highest on epidermal Langerhans cells (LCs) and can be upregulated by TGFβ (67). Furthermore, it has been shown that mutations in DC-HIL result in compromised ocular immunity and aberrant adaptive immune responses to self-antigen (68). ILT3 has similar properties, inducing anergy in CD4^+^ T cells, suppressing IFNγ-producing CD8^+^ cytotoxic T cells and inducing differentiation of CD8^+^ suppressor T cells (69). Additionally, it can function in antigen capture for processing and presentation to T cells (70). The cytoplasmic region of this receptor contains immunoreceptor tyrosine-based inhibitory motifs that negatively regulate the activation of APCs (70), reducing expression of co-stimulatory molecules and pro-inflammatory factors needed for T cell activation (71). Predominantly described as a potent co-inhibitory receptor on T cells, microglia express PD-1 during neurodegeneration. Microglial PD-1 is shown to limit infarct volume, recruitment of peripheral inflammatory cells, activation of microglia, and neurological deficits after experimentally induced middle cerebral artery occlusion (72). Engagement of PD-1 on LCs reduces pro-inflammatory cytokine production after TLR stimulation (73). Importantly, systemic anti-PD1 administration in an AD mouse model reduced pathology dependent on systemic IFNγ (25). However, since PD-1 gene expression is highly upregulated in the brain during neurodegeneration (Table 1), a second mode of action might occur within the brain parenchyma by modulating microglial PD-1. Microglia highly express the C-type lectin dectin-1 (encoded by *Clec7a*) and recognition of fungal β-glucans by dectin-1 represses cytokine production (74). Surprisingly, fungal infections have been found in post-mortem AD brain tissue (75), although the direct ligand for dectin-1 in the neurodegenerative brain remains to be identified. Recently, it has been show that dectin-1 signaling in microglia may be compensating for genetic loss of Trem-2, a risk factor associated with AD (76). Moreover, dectin-1-stimulated DCs and macrophages induce immunological tolerance (77). Stimulators of T cell functioning are also upregulated in microglia during neurodegeneration, including osteopontin (encoded by *Spp1*). In DCs, alternative translation of the same SPP1 mRNA generates two protein isoforms [intracellular (iOPN) and secreted (sOPN)] with distinct immunological effects. iOPN is shown to signal downstream of TLR7 and -9 and associates with MyD88, resulting in the activation of IRF7, induction of IFNα expression and polarization to Th1 T cell responses (78). Secreted osteopontin has been shown to affect intracranial T cell phenotype by inhibiting the release of IL-27 (79), sustaining the survival of CNS-reactive T cells and inducing a Th17 phenotype through CD44 expressed by infiltrating T cells (80). Importantly, a recent study has shown that CD44 is expressed particularly on CNS-infiltrating immune cells (81). This allows microglia to directly affect T cell polarization of incoming CNS-reactive T cells through secreted osteopontin (Figure 1). Since the appearance of CD11c^+^ microglia is prevalent neurodegenerative diseases, it is important to note that CD11c (*Itgax*) itself has previously been shown to inhibit TLR-mediated signaling *via* DAP12 in macrophages (82). In conclusion, microglia highly express the machinery that directly and indirectly affects T cell functioning, displaying a suppressive phenotype. In line with this, a microarray study on isolated microglia from the GL261 syngeneic glioblastoma model shows upregulation of the same gene set (65). Indeed, this model is highly enriched in regulatory T cells and immunosuppressive microglia (83). In parallel, gene expression analysis of glioblastoma patient tissue shows upregulation of these genes in tumor tissue (Table 1). The immunological response to neurodegenerative disease has previously been postulated to resemble tumor immunology (84). This suggests that microglia during neurodegenerative disease share similarities with tumor-associated microglia from a distinct immunosuppressive microenvironment.

It is perhaps surprising that the damage-associated microglia in mouse models of neurodegenerative disease express proteins capable of inducing T cell suppression/tolerance. However, their complete role in the neurodegenerative process still needs to be elucidated. Now that microglia are known to express modulators of T cell functioning, how does the interaction come about?

## Microglia-T Cell Crosstalk

### Two-Step Compartmentalized Antigen Presentation

Antigen presentation of draining CNS-derived antigens leads to T cell activation and infiltration in the brain (6). However, once the T cells arrive in the brain parenchyma, less is known about their fate and the signals they require to perform their function at the site of inflammation or neuronal damage. Early studies on antigen presentation by microglia have shown that microglia are inefficient activators of T cells and induce anergy or tolerance in T cells (85). However, under certain experimental conditions microglia are able to stimulate CD4^+^ and CD8^+^ T cells. For example, microglia are able to present antigen to T cells after exposure to IFNγ *in vivo* and *in vitro*, leading to the induction of regulatory T cells in an MHCII/CD86-dependent manner (86, 87). While there is little evidence that microglia migrate out of the brain to present CNS-derived antigen to naïve T cells in lymph nodes (see Box 1), they are able to modulate T cell responses once T cells are primed and enter the brain. Support for this two-step control of CNS-reactive T cells comes from an elegant study on CD4^+^ T cell-mediated neuroprotection after facial nerve injury (9). WT bone marrow (BM) chimeric animals that receive MHCII-KO BM after irradiation contain normal MHCII^+^ microglia, but no peripheral MHCII^+^ APCs. After facial nerve injury, no neuroprotection was observed, suggesting that peripheral MHCII^+^ APCs are needed to drive CD4^+^ T cell-mediated neuroprotection (9). Conversely, MHCII-KO animals that receive WT BM have MHCII^−^ microglia and MHCII^+^ APCs. Since these animals are devoid of CD4^+^ T cells, they were additionally reconstituted with either antigen-specific or antigen-unspecific CD4^+^ T cells. After facial nerve injury, again no neuroprotection was observed, suggesting that local radio-resistant MHCII^+^ APCs (i.e., microglia) are needed to drive CD4^+^ T cell-mediated neuroprotection (9). Importantly, only when pre-activated CD4^+^ T cells from facial nerve draining lymph nodes were adoptively transferred to the BM chimera animal, where MHCII-expression is confined to microglia, neuroprotection was restored (9). This suggests that CD4^+^ T cell-mediated neuroprotection is first initiated by peripheral APCs but needs a second local restimulation by MHCII^+^ microglia to drive antigen-specific neuroprotection. This compartmentalized control of T cell functioning targeted at the CNS could have significant consequences for neurodegenerative disease, since (1) activation of CNS-reactive T cells can be influenced and occur systemically, before clinical pathology and (2) local microglial control of CNS-reactive T cells requires cognate antigen engagement of MHCII:peptide complexes and specific TCRs. Unfortunately, similar elaborate studies to assess microglial antigen presentation and T cell control during neurodegenerative disease are currently lacking.

### Colocalization of Microglia and T Cells and the Outcome of Their Dialog

For two cell types to interact, their location in the brain should match and in the EAE mouse model of MS, myelin-specific T cells have been found in direct contact with lesion-reactive IL1β^+^ microglia (88). In EAE, a study of the kinetics of myelin uptake by CNS-resident cells suggests that microglia are the first cell type to modulate T cell responses inside the brain, before peripheral APCs arrive (89). However, there is a strong correlation between monocyte infiltration and progression of the clinical stage of EAE (90). Distinct differences in myeloid and monocytic population of APCs have been shown to differentially affect pathology during neurodegeneration (91). It has therefore been hypothesized that during MS and EAE, microglia are immune suppressive toward T cells, while peripheral immune infiltrates are causing T cell infiltration, activation, pathogenesis, and injury resolution (92). In brain tissue of PD patients, the CD8^+^ T cell population is increased and found in close proximity to activated microglia (16). In the MPTP mouse model of PD, microglia become MHCII-positive after phagocytosis of neurons and are in direct contact with infiltrating T cells (93). In the brains of AD patients, T cells can be found closely localized to microglia (94). And, transition into AD dementia correlates with increased MHCII^+^ microglia-mediated immunity and paralleled decrease in T cell number (41).

While their interaction seems apparent, the evidence whether MHCII-dependent antigen presentation is needed *in vivo* to cause neurodegeneration remains to be demonstrated. In the PD mouse model, α-synuclein induces microglial MHCII expression, antigen processing and presentation to CD4^+^ T cells (18). Knockout of MHCII in a mouse model of α-synuclein-dependent PD-like pathology resulted in reduced microglial activation and degeneration of dopaminergic neurons (18). Although α-synuclein-stimulated microglia presented the antigen to T cells *in vitro*, the precise role of MHCII^+^ microglia *in vivo* remains uncertain, since both MHCII and CD4^+^ T cells are absent in this model. Using cultures of primary microglia, Monsonego and colleagues showed that IFNγ enhanced clearance of Aβ, T cell motility, and microglia-T cell immunological synapse formation (95). In the 5XFAD mouse model of AD, the microglial phenotype switches toward a pro-inflammatory phenotype when T-, B-, and NK cells are genetically deleted (96). These data suggest that there may be differential outcomes of microglia–T cell interactions depending on either phenotype. In a PD mouse model, regulatory T cells have been shown to act directly on activated microglia, suppressing ROS production and NF-κB activation in both a soluble and cell-contact-dependent manner (19, 97). In the early phase of ALS-like symptoms in SOD1 transgenic mice, regulatory T cells are thought to actively contribute to neuroprotection by switching the phenotype of resident microglia (31). Also, infiltrates of the spinal cords of SOD1 transgenic mice showed increased amounts of CD4^+^ and CD8^+^ T cells and a time-dependent correlation between leukocyte infiltration and microglial expression profiles of antigen presentation machinery (63). Summarizing, CD4^+^ T cells infiltrate the brain during neurodegeneration and affect disease outcome, although it is still unclear how T cells orchestrate neurodegeneration and -protection.

### Microglia Subsets and the Damage-Associated Microglia

Whether subsets of microglia exist that perform specialized functions remains ambiguous. Recent studies have described a group of innate CD11c^+^ microglia that shows phenotypic and functional similarities with DCs (98, 99). During choriomeningitis virus (LCMV) infection, CD11c^+^ microglia have been shown to directly interact with CD8^+^ and CD4^+^ T cells *in vivo*, resulting in the purging of persistent brain infection and the generation of CNS-resident CD8^+^ memory T cells (100). The appearance of this CD11c^+^ microglia subset is widespread in murine models of neurodegeneration (Table 1) and may signify a phenotype that allows for interactions with infiltrating T cells. CD11c^+^ microglia were shown to express no pro-inflammatory cytokines and induced lower levels of Th1 and Th17 cytokines in T cells compared to CD11c^−^ microglia (99). This finding is substantiated by transcriptomics analysis of CD11c^+^ microglia isolated from a mouse model of AD (61). A more recent elegant study using single cell transcriptomics of isolated microglia during neurodegeneration attributes many genes included in Table 1 to a damage-associated microglia subset (101). Microglia isolated from models of neurodegeneration with this same damage-associated phenotype have recently been shown to directly affect antigen-specific T cell proliferation *in vitro* (48). Somewhat surprisingly, it was shown that microglia with this phenotype were less capable of suppressing T cell proliferation in an Apoe-dependent manner. While this is in apparent contrast with the upregulated genes as described above, it clearly shows that microglia from neurodegenerative diseases affect T cells in an activation- and antigen-dependent manner. Until now, research on antigen presentation by microglia has often been focused on paradigms that promote T cell activation, rather than prevent or suppress T cell activation. In the periphery, this latter feature is paramount for APCs to maintain immune homeostasis and prevent auto-immunity to self-proteins. Indeed, it would make sense that microglia exhibit similar characteristics to limit damage by self-reactive T cells in the CNS, preventing unrepairable neurodegeneration.

## Time-Dependent Changes in Microglia–T Cell Interactions and Consequences for Therapy

Most studies on mouse models of neurodegeneration suggest a time-dependent shift in CNS-reactive adaptive immunity. In this regard, a recent study by Keren-Shaul and colleagues has suggested a Trem-2 dependent two-step activation mechanism of damage-associated microglia, which involves the up- and downregulation of critical T cell modulators (101). Supplemented with data from Krasemann and collegues that the Trem2–ApoE axis affects the microglia-T cell interaction, it will be interesting to identify the direct mechanisms that influence this interaction over time. Indeed, the physiology of both T cell and microglia is changed with age, and it is not hard to imagine that an immunological equilibrium between parenchymal microglia, CNS-associated APCs and T cells is disturbed over time and contributes to neurodegenerative disease. Furthermore, the immunological dynamics of a human lifetime including systemic inflammation and commensal microbiota are known to impact the total immune system and may be causal to disease onset.

As a result of microglia–T cell crosstalk, changes in one of the two populations are likely to affect the phenotype of the other. Indeed, therapies that affect T cells during neurodegenerative diseases can indirectly affect microglial phenotypes and vice versa. This is evidenced by several studies using glatiramer acetate (GA) vaccination to change the dominant phenotype of CNS-infiltrating T cells. GA immunization generates a CNS-reactive T cell response that is protective in mouse models of neuronal injury (102), MS (103), AD (104), and PD (105). Most notably, the generated T cell response against CNS antigens leads to attenuated microglial activation in all of these studies. This suggests that the efficacy of T cell-modifying agents may be mediated through changes in the interaction with microglia. Conversely, changing microglial phenotype may affect T cell functioning during neurodegeneration. For example, through pharmacological inhibition of microglial proliferation in AD mice, markers for T cell infiltration were markedly reduced (64), including CD30 ligand, which is mainly expressed by activated T cells.

## Future Perspectives

An important challenge will be the identification of the repertoire of antigens being presented on MHC class II by microglia. Current methods to identify the peptide repertoire utilize mass spectrometry of purified MHC-peptide complexes, which is often hampered by the high amount of MHC^+^ cells needed. What sets the CNS apart from peripheral tissues is the lack of a parenchymal DC that takes up local antigen and migrates to draining lymph nodes for presentation, instead relying on antigen drainage to DCs outside the brain. Most of the self-proteins that accumulate during neurodegenerative diseases have aberrant post-translational modifications that result in reduced solubility. This could have two important consequences: (1) the insoluble antigen is unable to drain to secondary lymphoid structures where T cells are primed and (2) the insoluble antigen may harbor neo-antigens that can be presented by microglia on MHCII. As a result, microglia could present modified antigens to which no T cells are generated, creating an antigen mismatch in the brain that otherwise would have enabled microglia to interact with T cells. In this scenario, patients may benefit not only from protective T cell phenotypes but also protective or mimicked antigen specificity of the T cell.

Thus, immunotherapeutic strategies that aim to change the CNS inflammatory environment are likely to affect both T cells and microglia, which are engaged in an intimate tango, whether through soluble mediators or *via* contact-dependent interactions.

## Author Contributions

SS was involved in the writing, reading literature, design of the figure. DG-N and JJGV were involved in the supervision of the writing and content of the review. YK was inolvend in the overall supervision of the review and editing of the manuscript.

## Conflict of Interest Statement

The authors declare that the research was conducted in the absence of any commercial or financial relationships that could be construed as a potential conflict of interest.
